# Magic Mathematical Relationships for Nanoclusters—Errata and Addendum

**DOI:** 10.1186/s11671-019-3115-7

**Published:** 2019-08-27

**Authors:** Forrest H. Kaatz, Adhemar Bultheel

**Affiliations:** 1 Mesalands Community College, 911 South 10th Street, Tucumcari, 88401 NM USA; 2 Dept. Computer Sci., KU Leuven, Celestijnenlaan 200A, Heverlee, 3001 Belgium

**Keywords:** Nanoclusters, Topological indices, Coordination, Magic numbers

## Abstract

We correct magic formulas for body centered cubic (bcc) structures. The logical rational for this is further corroborated by calculations of the radial distribution function (RDF) for several crystal structures. We add results for truncated cubes which may be found in nature.

## Introduction

We recently presented magic formulas for several crystal nanoclusters [1]. However, it is known to crystallographers that bcc structures have a bulk coordination of eight. The RDF determines the nearest neighbor peaks from a central point, and the integrated peak intensity reflects the corresponding coordination for those neighbors. We use an established method [2] to calculate the RDF for several crystals. Since ideal bcc cubes have coordination *cn*=1, we provide results for truncated bcc and face centered cubic (fcc) clusters.

## Main Text

In reviewing the many magic formulas appearing in [1], it occurred to us that equation (1), which defines the adjacency matrix, depends on the crystal structure.

1 $$ \mathbf{A}(i,j)=\left\{\begin{array}{ll} 1& \text{if}\ r_{ij}< r_{c}\ \text{and}\ i\ne j\\ 0& \text{otherwise} \end{array}\right.  $$

Here, *r*_*ij*_ is the Euclidean distance between atom *i* and atom *j*. While it is true that *r*_*c*_=1.32·*r*_min_ is necessary for the different bond lengths in the dodecahedral structure, for the bcc structure, this is not the case. We have calculated [2] the RDF for selected structures, and some of the nearest neighbors are tabulated below (Table 1). The RDF has peak locations at neighbor sites, and the integrated intensity of the corresponding peak gives the coordination. We normalize the peaks in *R*(*r*) by dividing by the first peak, thus the peak locations become dimensionless. As the table indicates, bcc structures have $r_{c} = 2/\sqrt {3} \cdot r_{\text {min}} \approx 1.15 \cdot r_{\text {min}}$, which means the adjacency matrix must be changed, and thus the magic formulas. Note that neighbor peaks are not the same as shells, which give rise to the “magic numbers.” The dodecahedron is a complicated case, where the third neighbors appear at *r*_2_=1.31·*r*_min_. This case is challenging, and requires more analysis, which is in progress. The corrected bcc results are shown below (Tables 2, 3, 4, 5 and 6). These results agree with those in van Hardeveld and Hartog [3] if one shifts the index by one, i.e., we use the sequence 0, 1, 2... and they use 1, 2, 3... as their sequence. While perfect cubes may be of interest mathematically, they are not likely to appear in nature, due to single bonds at the corners. We have therefore generated truncated bcc and fcc cubes with the corners removed and their results are included in (Tables 7 and 8). The magic formulas of the indices for selected clusters are summarized in Table 9.

**Table 1:** Neighbor peaks in the normalized RDF for several structures

Structure	2nd nearest neighbor	3rd nearest neighbor	4th nearest neighbor
FCC	1.41	1.73	2.00
BCC	1.15	1.64	1.91
Hexagonal	1.41	1.73	1.91
Diamond	1.64	1.91	2.31
Simple cubic	1.41	1.73	2.00
Tetrahedron	1.41	1.73	2.00
Decahedron	1.41	1.73	1.91
Icosahedron	1.41	1.69	1.91
Dodecahedron	1.17	1.31	1.37

**Table 2:** Magic formulas for the bcc cube

	bcc cube	
	Atoms	2*n*^3^+3*n*^2^+3*n*+1, *n*≥1
	Bonds	8*n*^3^, *n*≥1
	*cn*=1	8, *n*≥1
	*cn*=2	12*n*−12, *n*≥1
	*cn*=4	6*n*^2^−12*n*+6, *n*≥1
bcc cube *n*=3	*cn*=8	2*n*^3^−3*n*^2^+3*n*−1, *n*≥1

**Table 3:** Magic formulas for the bcc octahedron

	bcc octahedron	
	Atoms	$\frac {8}{3}n^{3}+6n^{2}+\frac {16}{3}n+1,~n\ge 1$
	Bonds	$\frac {32}{3}n^{3}+12n^{2}+\frac {28}{3}n,~n\ge 1$
	*cn*=4	4*n*^2^+4*n*+6, *n*≥1
	*cn*=6	12*n*−12, *n*≥1
	*cn*=7	8*n*^2^−16*n*+8, *n*≥1
bcc octahedron *n*=3	*cn*=8	$\frac {8}{3}n^{3}-6n^{2}+\frac {16}{3}n-1,~n\ge {1}$

**Table 4:** Magic formulas for the bcc truncated octahedron

	bcc truncated octahedron	
	*n* odd	
	Atoms	$8n^{3}+\frac {9}{2}n^{2} +\frac {5}{2},~n\ge 1$ odd
	Bonds	32*n*^3^−6*n*^2^+6, *n*≥1 odd
	*cn*=4	9*n*^2^+5,*n*≥1 odd
	*cn*=6	6*n*−6, *n*≥1 odd
	*cn*=7	12*n*^2^−12*n, n*≥1 odd
	*cn*=8	$8n^{3}-\frac {33}{2}n^{2}+6n+\frac {7}{2},~n\ge {1}$ odd
	*n* even	
	Atoms	$8n^{3}+\frac {9}{2}n^{2} +3n+1,~n\ge 2$ even
	Bonds	32*n*^3^−6*n*^2^, *n*≥2 even
	*cn*=2	6n+12, *n*≥2 even
	*cn*=3	12*n*−24, *n*≥2 even
	*cn*=4	9*n*^2^−18*n*+14,*n*≥2 even
	*cn*=6	6*n, n*≥2 even
	*cn*=7	12*n*^2^−12*n, n*≥2 even
bcc truncated octahedron *n*=2	*cn*=8	$8n^{3}-\frac {33}{2}n^{2}+9n-1,~n\ge {2}$ even

**Table 5:** Magic formulas for the bcc rhombic dodecahedron

	bcc rhombic dodecahedron	
	Atoms	4*n*^3^+6*n*^2^+4*n*+1, *n*≥1
	Bonds	16*n*^3^+12*n*^2^+4*n*, *n*≥1
	*cn*=4	14, *n* ≥1
	*cn*=5	24*n*−24 *n* ≥1
	*cn*=6	12*n*^2^−24*n*+12, *n* ≥1
bcc rhombic dodecahedron *n*=2	*cn*=8	4*n*^3^−6*n*^2^+4*n*−1, *n* ≥1

**Table 6:** Magic formulas for the bcc cuboctahedron

	bcc cuboctahedron	
	Atoms	$\frac {5}{3}n^{3}+7n^{2}+\frac {34}{3}n+7,~n\ge 1$ odd
		$\frac {5}{3}n^{3}+7n^{2}+\frac {25}{3}n+1,~n\ge 2$ even
	Bonds	$\frac {20}{3}n^{3}+19n^{2}+\frac {64}{3}n+9,n\ge 1$ odd
		$\frac {20}{3}n^{3}+19n^{2}+\frac {46}{3}n,~n\ge 2$ even
	*cn*=2	12, *n*≥1 odd; 0, *n* even
	*cn*=3	12*n*−12, *n*≥1 odd; 0, *n* even
	*cn*=4	4*n*^2^−4*n*+6, *n*≥1 odd
		4*n*^2^+8*n*, *n*≥2 even
	*cn*=6	0, *n*≥1 odd; 12, *n* even
	*cn*=7	2*n*^2^+4*n*+2, *n*≥1 odd
		2*n*^2^+4*n*−16, *n*≥2 even
	*cn*=8	$\frac {5}{3}n^{3}+1n^{2}-\frac {2}{3}n-1,~n\ge {1}$ odd
bcc cuboctahedron *n*=2		$\frac {5}{3}n^{3}+1n^{2}-\frac {11}{3}n+5,~n\ge {2}$ even

**Table 7:** Magic formulas for the bcc truncated cube

	bcc truncated cube	
	Atoms	2*n*^3^+3*n*^2^+3*n*−7, *n*≥2
	Bonds	8*n*^3^−8, *n*≥2
	*cn*=2	12*n*−12, *n*≥2
	*cn*=4	6*n*^2^−12*n*+6, *n*≥2
	*cn*=7	8, *n*≥2
truncated bcc cube *n*=3	*cn*=8	2*n*^3^−3*n*^2^+3*n*−9, *n*≥2

**Table 8:** Magic formulas for the fcc truncated cube

	fcc truncated cube	
	Atoms	4*n*^3^+6*n*^2^+3*n*−7, *n*≥2
	Bonds	24*n*^3^+12*n*^2^−24, *n*≥2
	*cn*=5	12*n*−12, *n*≥2
	*cn*=7	24, *n*≥2
	*cn*=8	12*n*^2^−12*n*−18, *n*≥2
truncated fcc cube *n*=3	*cn*=12	4*n*^3^−6*n*^2^+3*n*−1, *n*≥2

**Table 9:** Magic topological formulas for BCC and FCC clusters

Index	Formula
bcc cube	
Wiener	$\frac {76}{35}n^{7} + \frac {38}{5}n^{6} + \frac {74}{5}n^{5} + 18n^{4} + \frac {206}{15}n^{3}+\frac {32}{5}n^{2}+\frac {136}{105}n$
Reverse Wiener	$\frac {64}{35}n^{7} + \frac {22}{5}n^{6} + \frac {31}{5}n^{5} + 2n^{4} - \frac {26}{15}n^{3}-\frac {17}{5}n^{2}-\frac {136}{105}n$
HyperWiener	$\frac {47}{35}n^{8}+\frac {226}{35}n^{7} + \frac {469}{30}n^{6} + \frac {241}{10}n^{5} + \frac {119}{5}n^{4} + \frac {149}{10}n^{3}+\frac {1097}{210}n^{2}+\frac {19}{35}n$
Szeged	NA
bcc rhombic dodecahedron	
Wiener	$\frac {76}{7}n^{7} + 38n^{6} + \frac {302}{5}n^{5} + 56n^{4} + \frac {94}{3}n^{3}+10n^{2}+\frac {148}{105}n$
Reverse Wiener	$\frac {148}{7}n^{7} + 58n^{6} + \frac {378}{5}n^{5} + 48n^{4} + \frac {38}{3}n^{3}-2n^{2}-\frac {148}{105}n$
HyperWiener	$\frac {359}{42}n^{8}+\frac {832}{21}n^{7} + \frac {1217}{15}n^{6} + \frac {1454}{15}n^{5} + 73n^{4} + \frac {103}{3}n^{3}+\frac {1957}{210}n^{2}+\frac {39}{35}n$
Szeged	$\frac {4637}{105}n^{9}+\frac {15655}{84}n^{8} + \frac {7661}{21}n^{7}+\frac {2615}{6}n^{6} + \frac {5194}{15}n^{5} + \frac {2245}{12}n^{4} + \frac {1412}{21}n^{3}+\frac {103}{7}n^{2}+\frac {32}{21}n$
bcc truncated cube	
Wiener	$\frac {76}{35}n^{7} + \frac {38}{5}n^{6} + \frac {74}{5}n^{5} - 6n^{4} - \frac {394}{15}n^{3}-\frac {168}{5}n^{2}+\frac {4336}{105}n$
Reverse Wiener	$\frac {64}{35}n^{7} + \frac {22}{5}n^{6} + \frac {31}{5}n^{5} - 6n^{4} - \frac {146}{15}n^{3}-\frac {57}{5}n^{2}+\frac {1544}{105}n$
HyperWiener	$\frac {47}{35}n^{8}+\frac {226}{35}n^{7} + \frac {469}{30}n^{6} + \frac {49}{10}n^{5} - \frac {121}{5}n^{4} - \frac {1313}{30}n^{3}+\frac {4457}{210}n^{2}+\frac {1933}{105}n$
Szeged	NA
fcc truncated cube	
Wiener	$\frac {956}{105}n^{7} + \frac {478}{15}n^{6} + \frac {1357}{30}n^{5} + \frac {110}{3}n^{4} + \frac {589}{30}n^{3}+\frac {97}{15}n^{2}+\frac {36}{35}n$
Reverse Wiener	$\frac {1564}{105}n^{7} + \frac {602}{15}n^{6} + \frac {1343}{30}n^{5} + \frac {70}{3}n^{4} + \frac {43}{15}n^{3}-\frac {59}{30}n^{2}-\frac {36}{355}n$
HyperWiener	$\frac {59}{10}n^{8}+\frac {2956}{105}n^{7} + \frac {1089}{20}n^{6} + \frac {701}{12}n^{5} + \frac {817}{20}n^{4} + \frac {1153}{60}n^{3}+\frac {53}{10}n^{2}+\frac {5}{7}n$
Szeged	$\frac {14822}{945}n^{9}+\frac {2099}{35}n^{8} + \frac {30781}{315}n^{7}+\frac {941}{10}n^{6} + \frac {1073}{18}n^{5} + \frac {251}{10}n^{4} + \frac {12629}{1890}n^{3}+\frac {29}{35}n^{2}+\frac {32}{105}n$

## Conclusions

We have corrected magic formulas for bcc structures and added results from the RDF and for truncated bcc and fcc cubes.

## Data Availability

The dataset(s) supporting the conclusions of this article may be obtained from the corresponding author.

## Abbreviations

bcc: Body centered cubic
fcc: Face centered cubic
RDF: Radial distribution function

## References

[1] Kaatz FH, Bultheel A (2019). Magic mathematical relationships for nanoclusters. Nanoscale Res Lett.

[2] Juhas P, Farrow CL, Yang X, Knox KR, Billinge SJL (2015). Complex modeling: a strategy and software program for combining multiple information sources to solve ill posed structure and nanostructure inverse problems. Acta Cryst.

[3] van Hardeveld R, Hartog F (1969). The statistics of surface atoms and surface sites on metal clusters. Surf Sci.

